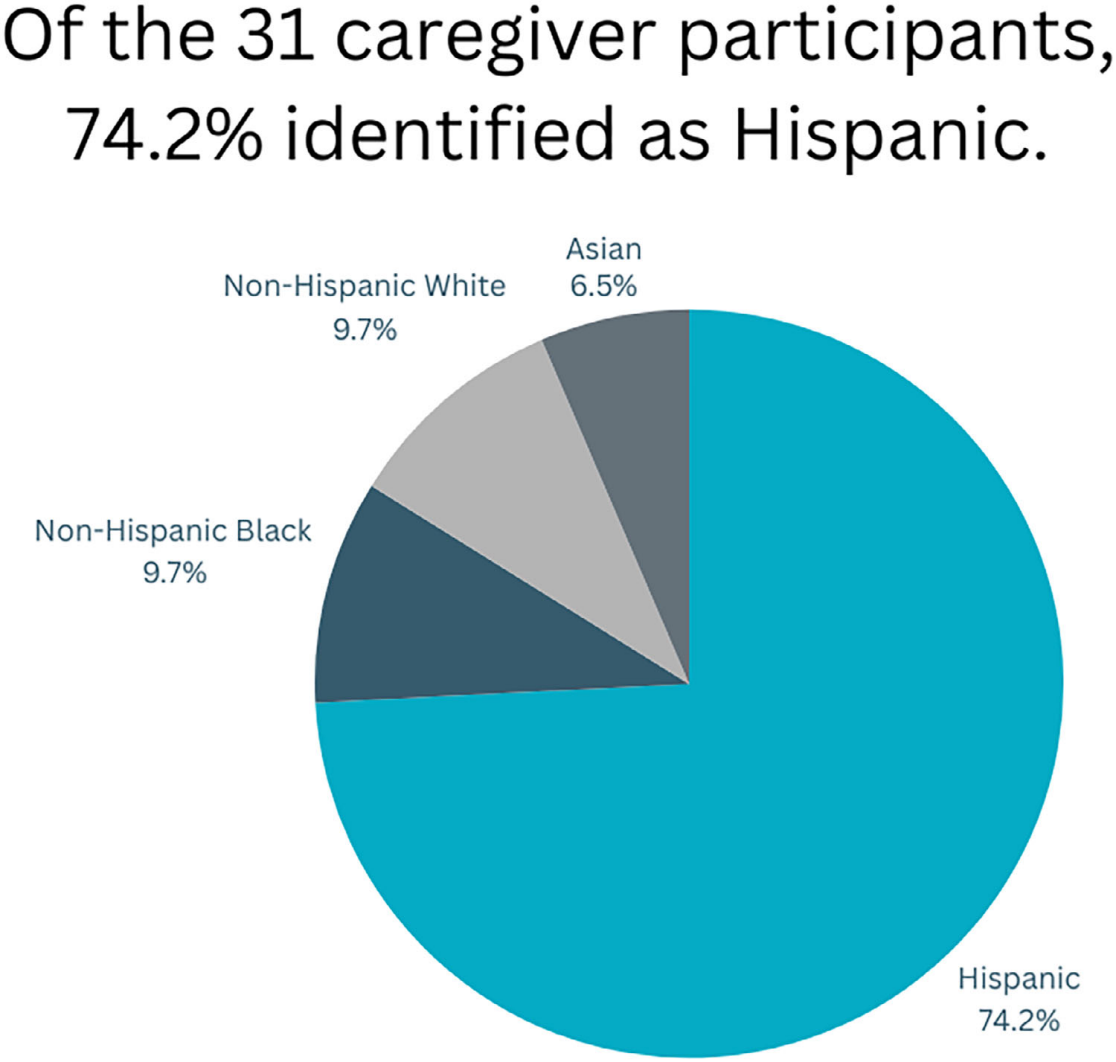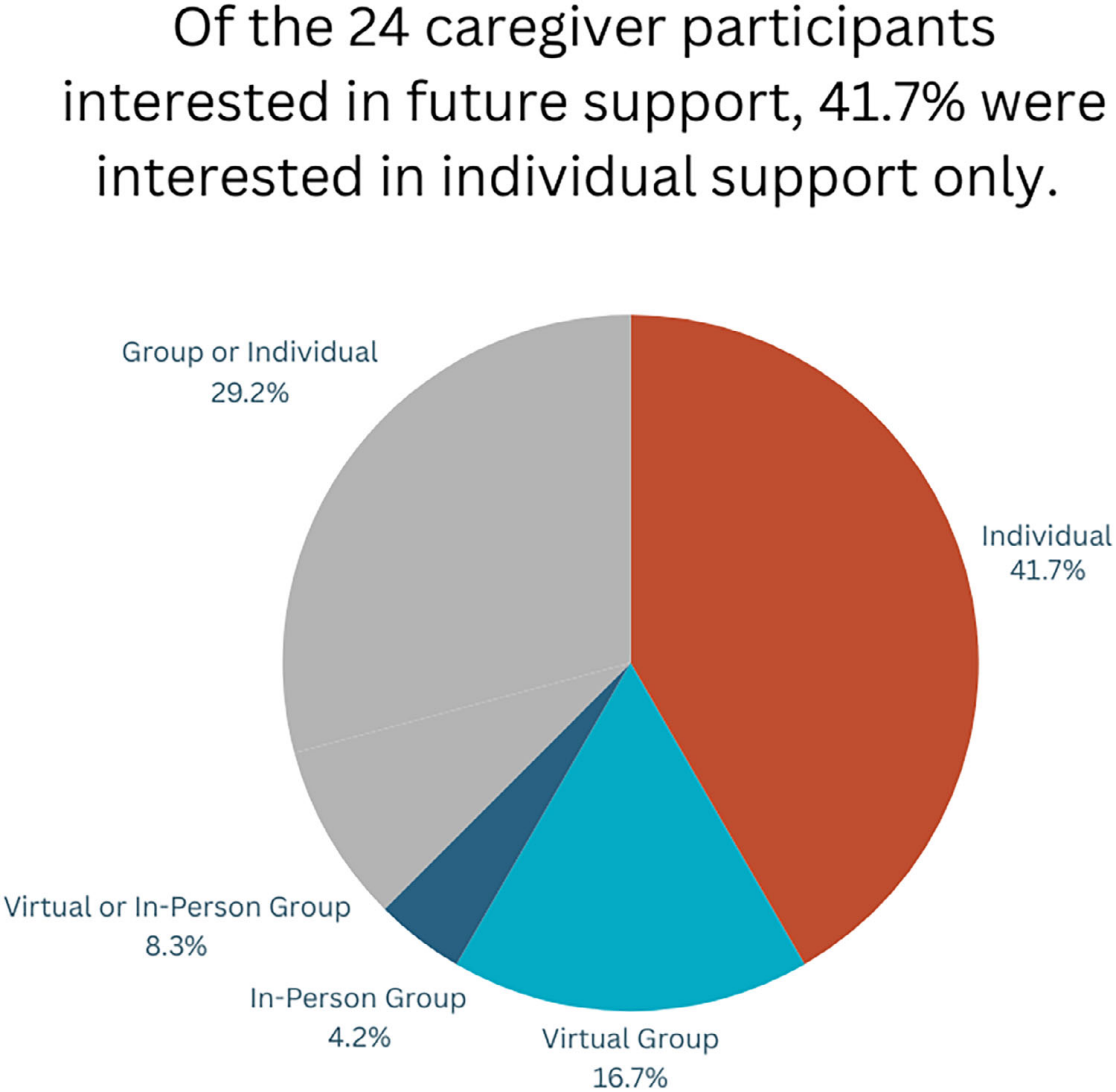# Uncovering the Needs of Culturally Diverse Caregivers of People Living With Dementia

**DOI:** 10.1002/alz70858_101208

**Published:** 2025-12-25

**Authors:** Noor Abi Rached, Morgan N McManus, Zuliany Lopez, Monique Lalane, Dominique Rennell, Sakina Ouedraogo Tall

**Affiliations:** ^1^ New York City Health and Hospitals Bellevue, New York, NY, USA; ^2^ NYU Grossman School of Medicine, New York City, NY, USA; ^3^ New York City Health and Hospitals Bellevue, New York City, NY, USA; ^4^ New York University Grossman School of Medicine, New York, NY, USA; ^5^ New York City Health and Hospitals, New York, NY, USA

## Abstract

**Background:**

Caregivers of people living with dementia (PLWD) face burdens and stressors despite the existence of caregiving resources in the community, which sometimes remain underutilized. The Telemedicine Cognitive Geriatric Program (TCGP) of the NYC Health and Hospitals Bellevue serves culturally diverse PLWD and provides caregiver support. We aim to understand the needs and preferences for support delivery of our caregiver population.

**Method:**

We retrospectively reviewed the services provided by the Social Worker (SW) to caregivers referred from the TCGP from May to December 2024. Using the electronic medical record, we identified all patients in the program. We administered a phone survey to caregivers, collected demographic information, past experiences with caregiver support, interest in future support, and preferences for support type (informational, emotional support, or tangible) and formats (individual vs. group, in person vs. virtual, open discussion vs. structured).

**Result:**

Of 85 patients receiving care, 26 have Mild Cognitive Impairment, 47 dementia (20 mild, 12 moderate, 15 severe). SW connected with 48 out of 54 caregivers identified. In addition to emotional support and psychoeducation, SW assisted 44% with obtaining an increase in home care services hours, 17% with acquiring durable medical equipment, connected 12% to transportation resources, and referred 64.5% to community programs.

Among 31 caregivers reached for the survey, 83.9% were female, 74.2% Hispanic, 9.7% African American, 9.7% Asian, 6.4% White. Most caregivers (61.3%) were children of the PLWD. About half (51.1%) received prior caregiver support, 77.4% expressed interest in future support with 75% preferring a mix of informational, emotional, and instrumental support. Most (41.7%) preferred individual support, 29.1% preferred group only, and 29.2% had no preference. Highlighted topics included managing behaviors, progression of dementia, and navigating home health and hospice services. Reasons for declining support were cultural differences, nature of familial bonds, and length of time and experience as a caregiver.

**Conclusion:**

These findings highlight the need for comprehensive and culturally responsive caregiver support programs. Providing practical assistance and addressing issues such as navigating healthcare services may improve engagement of culturally diverse caregivers. Future research should examine which components of these support programs influence caregiver well‐being and patient outcomes.